# Human Amniotic Epithelial Stem Cell Exosomes Regulate Chondrocyte Ferroptosis through ACTA2-AS1-Targeted Binding to ACSL4 for Osteoarthritis Intervention

**DOI:** 10.34133/research.0814

**Published:** 2025-08-08

**Authors:** Xiaofei Wang, Zhimin Wu, Lei Xu, Linbing Lou, Yuxia Yang, Jian Zhang, Haixiang Miao, Cunyi Xia, Zhiwei Peng, Dongsheng Yang, Zhiwen Tao, Xiangji Meng, Wenkang Liu, Meijuan Yuan, Jingcheng Wang, Wenyong Fei, Jihang Dai

**Affiliations:** ^1^ The Yangzhou School of Clinical Medicine of Dalian Medical University, Yangzhou 225001, Jiangsu, China.; ^2^ Department of Orthopedics, Northern Jiangsu People’s Hospital, Yangzhou 225001, Jiangsu, China.; ^3^ Northern Jiangsu People’s Hospital Affiliated to Yangzhou University, Yangzhou 225001, Jiangsu, China.; ^4^ Shandong Provincial Hospital Affiliated to Shandong First Medical University, Jinan 250021, Shandong, China.; ^5^ Department of Orthopedics, The Yangzhou Clinical Medical College of Xuzhou Medical University, Yangzhou 225001, Jiangsu, China.; ^6^ Sports Medicine Basic and Clinical Research Center of Yangzhou University, Yangzhou 225001, Jiangsu, China.

## Abstract

The inhibition of ferroptosis, a widespread form of nonapoptotic cell death, is considered a promising therapeutic approach for osteoarthritis (OA). Human amniotic epithelial stem cells (hAESCs) maintain multipotent differentiation potential, no tumorigenicity, low immunogenicity, and anti-inflammatory properties, rendering them highly biocompatible stem cells. Exosomes (Exo) are vesicular carriers for intercellular communication that participate importantly in regulating disease progression through paracrine signaling. In our study, under inflammatory stress conditions, actin alpha 2, smooth muscle antisense RNA1 (ACTA2-AS1) transcription was up-regulated in hAESCs, further delivered to chondrocytes via hAESC-derived Exo. Subsequently, ACTA2-AS1 could suppress ferroptosis in chondrocytes by facilitating the degradation of acyl-CoA synthetase long-chain family member 4 (ACSL4), a key regulator of ferroptosis, thereby modulating the progression of OA. In conclusion, for the first time, this study demonstrates the modulatory role of hAESC ACSL4 expression by releasing ACTA2-AS1-enriched Exo, leading to inhibited ferroptosis in chondrocytes and ultimately ameliorating OA progression. Thus, targeting Exo-mediated communication may offer novel therapeutic approaches for addressing OA linked to iron metabolism irregularities.

## Introduction

Osteoarthritis (OA) is a multifaceted degenerative condition characterized by articular cartilage deterioration and joint inflammation, leading to impaired joint function [1–3]. The prevalence of OA continues to rise steadily, particularly due to the aging population globally, imposing a substantial economic burden worldwide [4]. Symptomatic treatment remains the primary therapeutic option for OA, given its unclear pathogenesis and numerous undisclosed links. In order to enable the comprehensive management of OA, active studies have been conducted to unveil novel therapeutic targets [5,6]. Thus, this highlights the urgency of further investigation into the underlying mechanism of and suitable treatment modalities for OA.

Human pluripotent stem cells possess a robust unlimited self-renewal capacity to differentiate into derivatives of the 3 embryonic layers, rendering them attractive for cartilage regeneration and OA therapy [7]. Nonetheless, concerns related to their tumorigenicity, donor–recipient immune incompatibility, and ethical issues are existing challenges that impede the utilization of stem cells for OA treatment [8]. Significantly, human amniotic epithelial stem cells (hAESCs) have emerged recently as a promising alternative due to their multipotent differentiation potential, nontumorigenic nature, low immunogenicity, and anti-inflammatory properties. While circumventing ethical dilemmas, hAESCs also exhibit pluripotent characteristics akin to those of embryonic stem cells [9–12]. Therefore, hAESCs may represent a favorable candidate for OA therapy. Nevertheless, there are no reports on the specific mechanism of hAESCs in OA treatment, so it is very promising and meaningful to study the treatment of OA with hAESCs.

The efficacy of stem cell intervention in OA has been highlighted by several clinical registration trials recently [13,14]. Stem cells can release exosomes (Exo), which act as “shuttle vesicles” transporting small molecules such as nucleic acids or proteins. Through molecular interactions with chondrocytes, Exo perform various functions, including hindering chondrocyte apoptosis and inflammatory responses, facilitating autophagy, promoting chondrocyte extracellular matrix (ECM) production, and modulating immune responses [15]. The beneficial effects of certain stem-cell-derived Exo have been uncovered in OA treatment [16]. Whether hAESCs can effectively mitigate OA progression and whether hAESCs have a specific effect on OA progression through their Exo need further study. Specifically, stem-cell-derived Exo can reinforce intercellular communication by transporting proteins, lipids, long noncoding RNA (lncRNA), circular RNA, and microRNA (miRNA). Numerous studies [17–21] have indicated the inhibitory role of stem-cell-derived Exo in OA progression by delivering miRNA to chondrocytes; however, research into lncRNA remains limited. hAESCs are widely recognized for their fetal origin, hypoimmunogenic properties, pluripotent differentiation capabilities, and their secretion of Exo (hAESC-derived Exo, hAESCs-Exo). As a cell-free therapeutic vector, they exhibit important transformative potential; however, the specific role of OA in this context remains to be elucidated. In addition, under a persistent inflammatory microenvironment, stem cells are commonly believed to experience compromised functionality. Nonetheless, stem cells exhibit notable resilience to inflammatory stress and may even augment their functionality in specific inflammatory microenvironments [22–24]. In combination with the above interpretation, the potential of Exo secreted by hAESCs in an inflammatory environment (IE-hAESCs-Exo) for immune regulation, anti-inflammatory effects, and the promotion of chondrocyte repair in OA warrants further investigation.

Recent studies have demonstrated a close association of various metabolic factors, such as hyperlipidemia and iron overload, with the onset of OA. For instance, ferroptosis and disrupted iron metabolism may contribute to the pathological manifestations of OA, including cartilage degradation, synovial damage, and subchondral bone sclerosis, in a direct or indirect manner [25–27]. Ferroptosis represents an iron-dependent regulated cell death mechanism that is propelled by iron-dependent excessive lipid peroxidation, with promising therapeutic implications for degenerative conditions [28]. Mounting evidence underscores the intricate interplay between OA, despite being classified as a degenerative disorder, and chronic inflammation, which significantly influences the severity of pain and disease progression. The persistent inflammatory milieu in the cartilage and joint space of OA stands out as a defining feature of the disease [29].

In our study, a hypothesis was proposed that hAESC-derived Exo could deliver lncRNA to chondrocytes, inhibiting ferroptosis via sensitive regulators and ultimately modulating OA progression. To identify key proteins or pathways involved in ferroptosis during OA, an integrated proteomic approach was employed to examine the metabolomic changes associated with ferroptosis in OA. Additionally, the binding of lncRNA to ferroptosis regulators was identified by RNA-immunoprecipitation and high-throughput sequencing (RIP-seq), aiming to trace targets, verify the mediation of the pathological link between ferroptosis and OA by hAESC-derived Exo, and identify specific targets. It is expected that hAESC-derived Exo may significantly inhibit the pathological process of ferroptosis in chondrocytes, thereby mitigating OA. Furthermore, these Exo may down-regulate acyl-CoA synthetase long-chain family member 4 (ACSL4) expression by delivering lncRNA ACTA2-AS1 (actin alpha 2, smooth muscle antisense RNA1) in inflammatory conditions, providing a molecular mechanism for regulating ferroptosis.

## Results

### hAESCs improved pathological changes in knee cartilage in rats

**Fig. 1.**
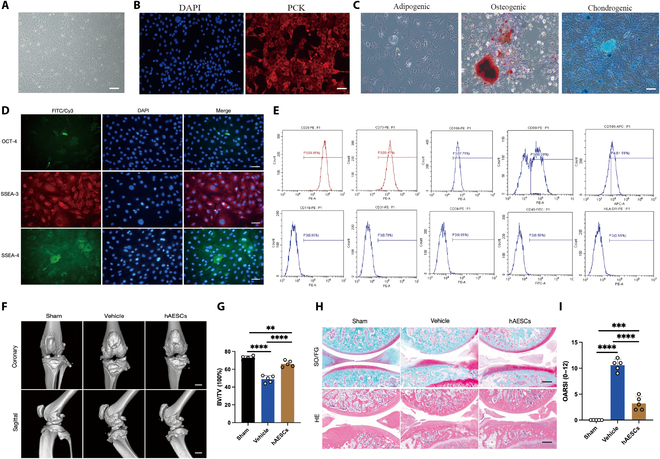
Human amniotic epithelial stem cells(hAESCs) improved pathological changes in knee cartilage in rats. (A) Morphology of third-generation hAESCs under microscopy. Scale bar: 20 μm. (B) Immunofluorescence staining reveals pancytokeratin (PCK) expression in hAESCs. Scale bar: 20 μm. (C) Microscopic analysis of hAESC-induced trilineage differentiation. Scale bar: 20 μm. (D) Immunofluorescence indicates the molecular marker expression of hAESCs on embryonic stem cells. Scale bar: 20 μm. (E) Flow cytometry analysis shows hAESC expression of markers related to mesenchymal and hematopoietic stem cells. (F) Micro-computed tomography (micro-CT) results of rat knee joints. Scale bar: 5 mm. (G) Bone volume fraction (BV/TV) statistical analysis (*n* = 5 per group). (H) Safranin O/Fast Green (SO/FG) and hematoxylin–eosin (HE) staining of rat knee joints at 6 weeks. Scale bar: 200 μm. (I) Osteoarthritis Research Society International (OARSI) score (*n* = 5 per group). All data are presented as mean ± SD. Statistical significance was defined as *P* < 0.05 (*P < 0.05; **P < 0.01; ***P < 0.001; ****P < 0.0001). DAPI, 4′,6-diamidino-2-phenylindole; FITC, fluorescein isothiocyanate.

Following the isolation and culture of hAESCs from amniotic membranes, morphological analysis revealed diverse cell shapes, including spindle, oval, and polygonal forms, with strong adhesion to the culture surface and rapid proliferation (Fig. 1A). Immunofluorescence confirmed the epithelial origin of hAESCs determined by pancytokeratin positivity (Fig. 1B). Then, hAESCs were induced to differentiate into osteoblasts, adipocytes, and chondrocytes to assess their differentiation potential. According to the results (Fig. 1C), lipid droplet O staining indicated red cytoplasmic staining in cells cultured in adipogenic medium. Eosin S staining showed calcium phosphate deposition in cells cultured in osteogenic medium, while alizarin blue staining demonstrated glycosaminoglycan production in cells cultured in chondrogenic medium. Further data analyses (Fig. 1D) reveal that hAESCs expressed key embryonic stem cell markers, including embryonic antigens SSEA-3 and SSEA-4, and a transcription factor, OCT-4, essential for maintaining pluripotency and self-renewal. To this end, it was confirmed that hAESCs exhibited multipotent differentiation. Furthermore, phenotypic characterization using flow cytometry indicated that hAESCs were detected with high levels of mesenchymal stem cell markers CD29, CD73, and CD166 but weakly positive CD90 and CD105, distinguishing them from typical mesenchymal stem cells. Notably, hematopoietic stem cell markers CD11b, CD31, CD34, and CD45 failed to express in hAESCs, with the detection of negative HLA-DR as well (Fig. 1E). These results supported a successful extraction of hAESCs from the amniotic membrane. To further explore the therapeutic effects of hAESCs on OA in vivo, a rat OA model was constructed using the destabilization of the medial meniscus (DMM), a well-recognized in vivo model. After operation, changes in articular cartilage and bone volume fraction (BV/TV) were assessed and analyzed using micro-computed tomography (micro-CT) (Fig. 1F). The hAESC-treated group exhibited a significant improvement in DMM-induced BV/TV (Fig. 1G). Meanwhile, histological analysis revealed severe joint surface degradation, ossification, and disrupted cartilage tide-line structure in the vehicle group, aligning with the typical pathology of OA. Conversely, the hAESC-treated group showed restored cartilage staining, distinct cartilage and subchondral bone boundaries, enhanced ECM synthesis, and recovered tidal lines (Fig. 1H). In addition, Osteoarthritis Research Society International (OARSI) scores indicated significant reduction in the hAESC-treated group compared to those in the vehicle group, suggesting notable improvements in bone mineral density (BMD) and structural integrity, as well as restored bone microarchitecture (Fig. 1I).

### hAESCs-Exo uptake of chondrocytes

**Fig. 2.**
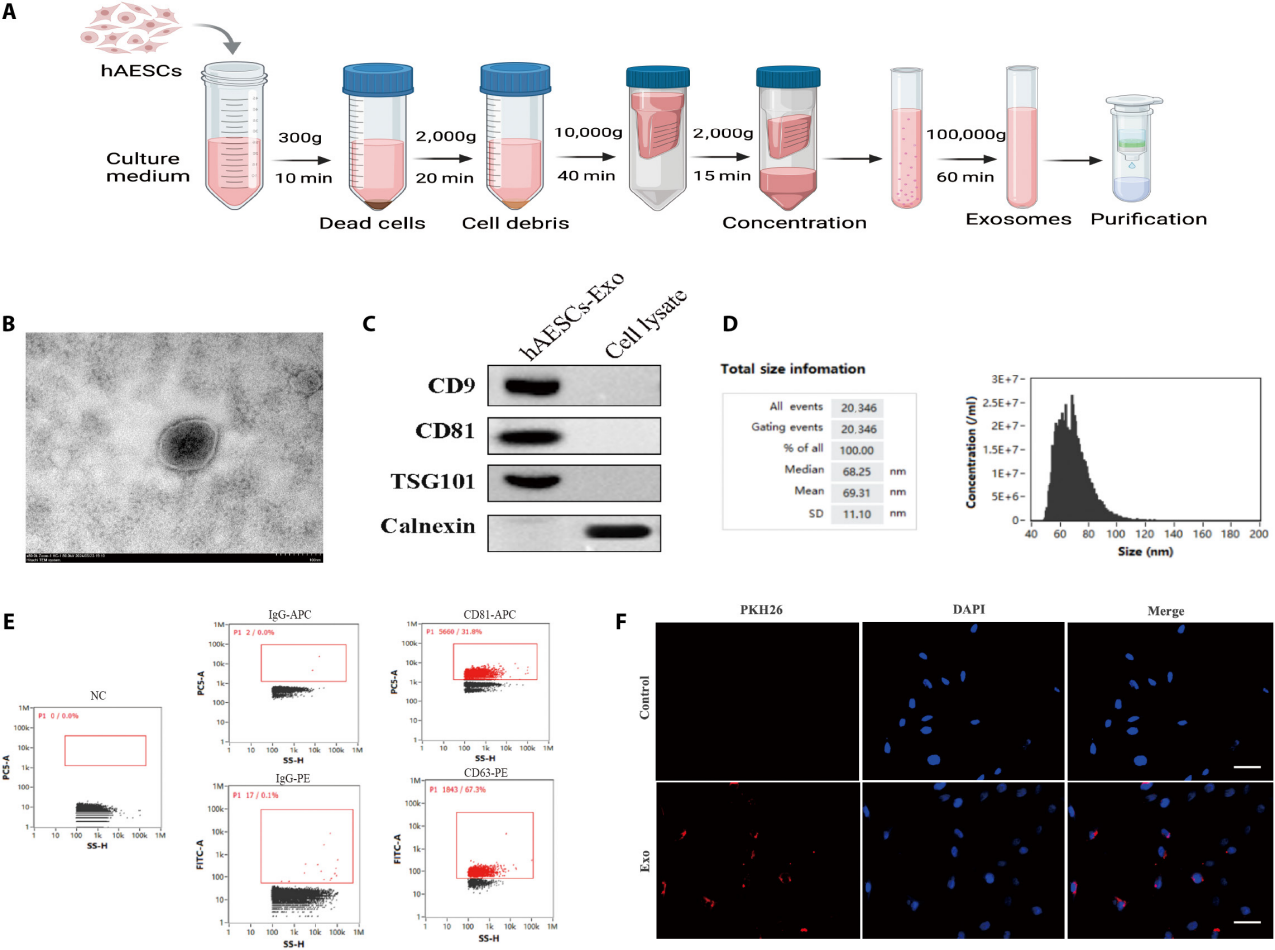
hAESC-derived Exo (hAESCs-Exo) uptake of chondrocytes. (A) Schematic diagram illustrating exosome extraction from hAESC supernatant via differential centrifugation. (B) Transmission electron microscopy reveals the morphology of hAESCs-Exo. Scale bar: 100 nm. (C) Western blot analysis identifies exosome marker protein expression. (D) Nanoparticle size analysis determines the size distribution of exosomes. (E) Flow cytometry assesses hAESCs-Exo marker antigens. (F) Fluorescence microscopy visualizes the uptake of PKH26-labeled hAESCs by chondrocytes. Scale bar: 20 μm.

Exo are accepted as a type of nanomaterials facilitating intercellular communication and can mediate paracrine effects. In this study, Exo were isolated from hAESC culture supernatant using ultrafiltration and differential ultracentrifugation (Fig. 2A). Transmission electron microscopy (TEM) revealed that the purified hAESC-derived Exo were spherical in shape with low-density centers (Fig. 2B). Western blot analysis confirmed the presence of exosomal markers CD9, CD81, and TSG101, while calnexin was absent in the Exo but present in cell lysates (Fig. 2C). Comprehensive bio-nanoparticle analysis verified that the particle size of Exo ranged between 30 and 150 nm (Fig. 2D). Flow cytometry analysis confirmed the presence of the marker antigens CD81 and CD63 in hAESCs-Exo (Fig. 2E), indicating successful purification and identification of these Exo. In addition, to assess the uptake of hAESCs-Exo by chondrocytes, human cartilage tissues were harvested to extract cells for 24 h of co-culture with PKH26-labeled hAESCs-Exo. Eventually, successful uptake of the labeled Exo by chondrocytes was confirmed by fluorescence microscopy (Fig. 2F).

### hAESCs-Exo regulated ferroptosis in human OA chondrocytes

**Fig. 3.**
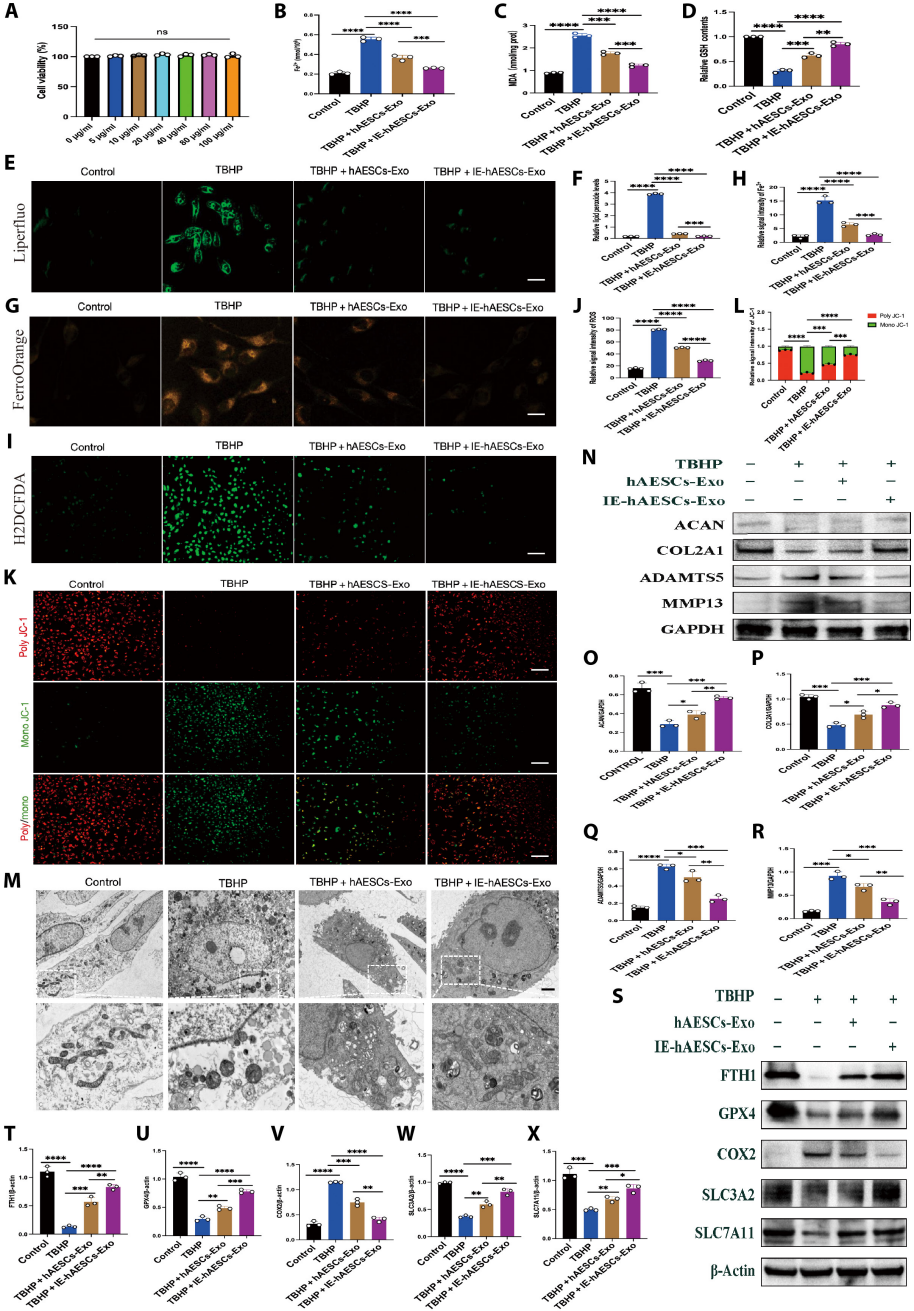
hAESCs-Exo regulated ferroptosis in human osteoarthritis (OA) chondrocytes. (A) Cell Counting Kit-8 (CCK-8) experiments evaluate exosome biosafety (*n* = 3 per group). (B) Cell ferrous colorimetry to detect the intracellular Fe^2+^ levels of chondrocytes (*n* = 3 per group). (C) Malondialdehyde (MDA) colorimetry to determine the MDA concentration in chondrocytes (*n* = 3 per group). (D) Glutathione (GSH) colorimetry to quantitatively determine the content of reduced GSH (*n* = 3 per group). (E) The Liperfluo probe detects the fluorescence expression of lipid peroxidation in chondrocytes. Scale bar: 20 μm. (F) Quantitative analysis of lipid peroxidation fluorescence intensity (*n* = 3 per group). (G) FerroOrange fluorescence detects changes in intracellular Fe^2+^ levels. Scale bar: 20 μm. (H) FerroOrange fluorescence detection quantitative analysis of intracellular Fe^2+^ fluorescence intensity (*n* = 3 per group). (I) H2DCFDA fluorescent probe detects reactive oxygen species (ROS) expression in chondrocytes. Scale bar: 50 μm. Quantitative analysis of the fluorescence intensity of the ROS expression of the H2DCFDA fluorescence probe (*n* = 3 per group). (J) Quantitative analysis of ROS expression using the H2DCFDA fluorescent probe (*n* = 3 per group). (K) JC-1 fluorescent probe assessment of mitochondrial membrane potential changes in chondrocytes. Scale bar: 50 μm. (L) Quantitative analysis of mitochondrial membrane potential alterations in chondrocytes (*n* = 3 per group). (M) Transmission electron microscopy examination of mitochondrial morphological changes in chondrocytes. Scale bar: 5 μm. (N to R) Western blot analysis and quantification of the effects of IE-hAESCs-Exo (exosomes secreted by hAESCs in an inflammatory environment) on extracellular matrix metabolic balance protein expression in chondrocytes (*n* = 3 per group). (S to X) Western blot analysis and quantification of IE-hAESCs-Exo effects on ferroptosis marker protein expression (*n* = 3 per group). All data are presented as mean ± SD. Statistical significance was defined as *P* < 0.05 (*P < 0.05; **P < 0.01; ***P < 0.001; ****P < 0.0001). TBHP, *tert-*butyl hydroperoxide.

Cell Counting Kit-8 (CCK-8) assay was utilized to clarify the impact of hAESCs-Exo on chondral toxicity and biosafety. At concentrations ranging from 0 to 100 μg/ml, hAESCs-Exo exposure for 24 h resulted in no significant changes in cell proliferation or death, suggesting negligible cytotoxicity (Fig. 3A). Cell survival/death assays corroborated these findings (Fig. S1). As described in our introduction, to investigate the regulatory effects of Exo secreted by hAESCs on OA in an inflammatory environment, we pretreated third-generation hAESCs with a complete medium containing 10 ng/ml IL-1β to induce inflammation. Once the cells reached 50% to 60% confluency, we replaced the complete medium with a serum-free medium devoid of Exo. The cells were then cultured for an additional 48 h, after which Exo were extracted from the collected cell supernatants. A subsequent experiment focused on examining the effects of hAESCs-Exo and IE-hAESCs-Exo on chondrocyte ferroptosis induced by *tert*-butyl hydroperoxide (TBHP) solution (50 μM). TBHP exposure led to apoptosis, elevated total reactive oxygen species (ROS), lipid peroxidation, and cell death in chondrocytes. However, TBHP-induced cell death was not mitigated after the application of apoptosis (ZVAD) and necroptosis (Necro) inhibitors [30,31]. Notably, IE-hAESCs-Exo significantly reduced intracellular Fe^2+^ and malondialdehyde (MDA) levels (Fig. 3B and C) but markedly increased glutathione (GSH) levels (Fig. 3D). We assessed the efficacy of the fluorescence probe Liperfluo in detecting lipid peroxidation. TBHP-induced lipid peroxidation was notably inhibited by both hAESCs-Exo and IE-hAESCs-Exo, with a more pronounced effect found by the latter one (Fig. 3E and F). Additionally, with the use of a novel fluorescent probe FerroOrange, which images Fe^2+^ in living cells, IE-hAESCs-Exo treatment resulted in a significant reduction in Fe^2+^ levels (Fig. 3G and H). Meanwhile, using an ROS fluorescence probe, IE-hAESCs-Exo effectively mitigated the TBHP-induced rise in intracellular ROS levels (Fig. 3I and J). Subsequently, it was noticed that TBHP treatment decreased mitochondrial membrane potential (MMP) in chondrocytes, leading to the dissociation of polymers into monomers and an increase in green fluorescence (single JC-1). IE-hAESCs-Exo treatment also increased red fluorescence (poly JC-1) obviously (Fig. 3K and L). TEM revealed characteristic mitochondrial death morphology in chondrocytes. In TBHP-induced OA chondrocytes, it was observed to have a ruptured outer mitochondrial membrane and diminished or absent mitochondrial cristae. Noticeably, IE-hAESCs-Exo treatment significantly ameliorated these mitochondrial morphological changes in the TBHP-induced OA model (Fig. 3M). Western blot analysis indicated enhanced catabolic responses of chondrocytes in this model, with significantly elevated expression of MMP13 and ADAMTS5, further intensifying the catabolic activity. Notable down-regulation was detected in ECM-related genes, such as ACAN and COL2A1. However, IE-hAESCs-Exo treatment effectively decreased MMP13 and ADAMTS5 protein levels while increasing ACAN and COL2A1 levels (Fig. 3N to R). In addition, IE-hAESCs-Exo treatment resulted in obvious down-regulation of ferroptosis-related marker genes, including the positive regulator COX2. In contrast, significant up-regulation was observed in the inhibitors of ferroptosis, such as FTH1, GPX4, SLC3A2, and SLC7A11 (Fig. 3S to X). Collectively, these findings strongly suggested that hAESCs-Exo interacted with ferroptosis to decelerate OA progression.

### hAESCs-Exo inhibited cartilage degeneration in a rat model of DMM-induced OA

**Fig. 4.**
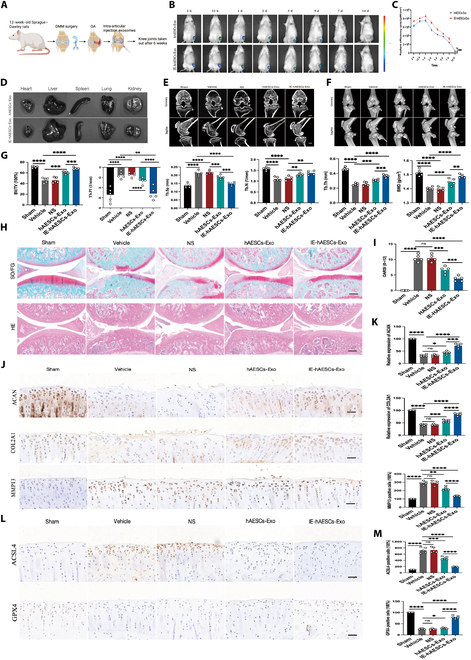
hAESCs-Exo inhibited cartilage degeneration in a rat model of destabilization of the medial meniscus (DMM)-induced OA. (A) Schematic of the knee exosome experiment in Sprague–Dawley rats. (B to D) PKH26 fluorescent labeling and in vivo imaging were used to assess exosome distribution dynamics and off-target risks (*n* = 3 per group). (E to G) Micro-CT was employed to observe and calculate changes in BV/TV, trabecular bone, and bone mineral density (BMD) (*n* = 5 per group). Scale bar: 5 mm. (H and I) SO/FG and HE staining, along with OARSI scoring, were conducted on knee joints at 6 weeks (*n* = 5 per group). Scale bar: 200 μm. (J and K) Immunohistochemistry and quantitative analysis of COL2A1, ACAN, and MMP13 expression in rat knee joints (*n* = 5 per group). Scale bar: 100 μm. (L and M) Immunohistochemistry was used to detect ACSL4 and GPX4 expression in knee joint cartilage, with subsequent quantitative analysis (*n* = 5 per group). Scale bar: 100 μm. All data are presented as mean ± SD. Statistical significance was defined as *P* < 0.05 (*P < 0.05; **P < 0.01; ***P < 0.001; ****P < 0.0001). NS, normal saline; Tb.Pf, trabecular pattern factor; Tb.Sp, trabecular separation; Tb.N, trabecular number; Tb.Th, trabecular thickness.

To investigate the impact of hAESCs on OA progression, a DMM-induced OA model was constructed in 12-week-old Sprague–Dawley rats by intra-articular administration of hAESCs-Exo or normal saline over a 6-week period (Fig. 4A). Our experimental protocol involved injecting PKH26-labeled hAESCs-Exo into rat knee joints and in vivo fluorescence imaging along with semiquantitative analysis. The results indicated that the Exo were rapidly enriched in the knee cavity of the rats, with fluorescence intensity peaking 1 d postinjection. By day 7, both sets of fluorescent signals had decreased significantly, returning to background levels by day 14. Notably, the contrast in fluorescence intensity between IE-hAESCs-Exo and hAESCs-Exo was significant. These findings suggest that the Exo derived from hAESCs following inflammatory pretreatment may enhance their retention in the joint (Fig. 4B and C). According to the live fluorescence imaging of various organs (e.g., heart, liver, spleen, lung, and kidney), Exo primarily targeted the joint, without significant off-target fluorescence in other organs (Fig. 4D). Furthermore, joint samples were collected for micro-CT examination. The subchondral bone morphology was observed to be significantly changed in the vehicle group, especially the production of a large number of osteophytes and the significantly reduced BV/TV. Significantly, IE-hAESCs-Exo effectively ameliorated these pathological changes and inhibited the changes in trabecular bone (trabecular pattern factor [Tb.Pf], trabecular separation [Tb.Sp], trabecular number [Tb.N], and trabecular thickness [Tb.Th]) and BMD in OA rats (Fig. 4E to G). Furthermore, Safranin O/Fast Green and hematoxylin–eosin staining indicated that intra-articular injection of IE-hAESCs-Exo effectively attenuated DMM-induced degeneration of articular and subchondral bones in OA rats (Fig. 4H and I). Immunohistochemistry (IHC) of articular cartilage revealed up-regulated expression of ACAN and COL2A1 and down-regulated production of MMP13 after hAESCs-Exo treatment (MMP13) (Fig. 4J and K). Additionally, IE-hAESCs-Exo notably suppressed the expression of ACSL4 in cartilage tissues and enhanced the expression of GPX4 (Fig. 4L and M). Altogether, both hAESCs-Exo and IE-hAESCs-Exo could impede cartilage aging in chondrocyte ferroptosis and OA models, with IE-hAESCs-Exo demonstrating superior efficacy. The observed changes in the histological profile of rats aligned with the outcomes of our in vitro analyses.

### Proteomics analysis revealed a significant enrichment of ACSL4 in human OA chondrocytes after IE-hAESCs-Exo treatment

**Fig. 5.**
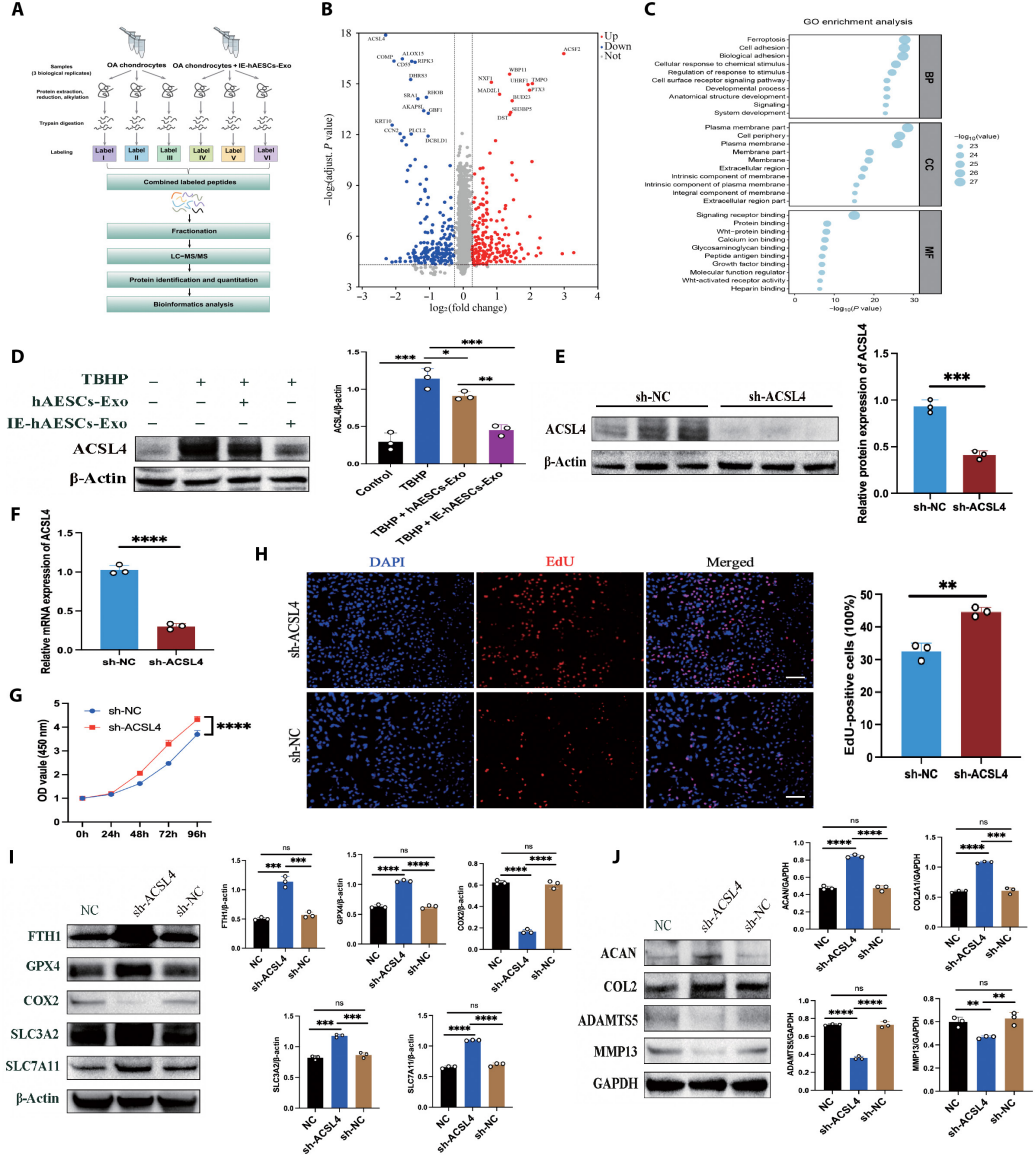
Proteomics analysis revealed a significant enrichment of ACSL4 in human OA chondrocytes after IE-hAESCs-Exo treatment. (A) Schematic of the proteomics sequencing workflow. (B) Volcano plot illustrating differentially expressed proteins pre- and postprocessing. (C) Kyoto Encyclopedia of Genes and Genomes (KEGG) pathway analysis revealing pathway inhibition. (D) Western blot analysis of ACSL4 expression changes and quantitative assessment (*n* = 3 per group) in OA chondrocytes induced by TBHP. (E) Western blot evaluation of sh-ACSL4 transfection efficiency in chondrocytes and quantitative analysis (*n* = 3 per group). (F) Quantitative real-time polymerase chain reaction (qRT-PCR) assessment of sh-ACSL4 transfection efficiency in chondrocytes (*n* = 3 per group). (G) CCK-8 assay measuring chondrocyte proliferation following sh-ACSL4 viral transfection. (H) 5-Ethynyl-2′-deoxyuridine (EdU) staining for the proliferation capacity assessment of chondrocytes post-sh-ACSL4 viral transfection, with quantitative analysis (*n* = 3 per group). Scale bar: 50 μm. (I) Western blot analysis of ferroptosis-related marker genes and quantitative assessment post-sh-ACSL4 (*n* = 3 per group). (J) Western blot analysis of chondrocyte matrix metabolism markers and quantitative assessment post-sh-ACSL4 (*n* = 3 per group). All data are presented as mean ± SD. Statistical significance was defined as *P* < 0.05 (*P < 0.05; **P < 0.01; ***P < 0.001; ****P < 0.0001). LC–MS/MS, liquid chromatography–tandem mass spectrometry; GO, Gene Ontology; BP, Biological Process; CC, Cellular Component; MF, Molecular Function; mRNA, messenger RNA; OD, optical density.

To investigate the potential mechanisms underlying the therapeutic efficacy of IE-hAESCs-Exo for the treatment of OA, human OA chondrocytes were analyzed by proteomic sequencing before and after the treatment of IE-hAESCs-Exo, and the differential changes in protein expression were compared between the 2 groups (Fig. 5A). IE-hAESCs-Exo treatment resulted in a significant down-regulation of ACSL4, a key regulator of ferroptosis (Fig. 5B). Furthermore, signaling pathway enrichment indicated a notable inhibition of the ferroptosis pathway (Fig. 5C). These findings suggest that IE-hAESCs-Exo might modulate OA progression by interfering with the ferroptosis process in chondrocytes. Previous research has linked ACSL4 to various metabolic disorders, highlighting its role as a sensitive ferroptosis regulator [32]. Therefore, through genetic and pharmacological means, suppression of ACSL4 expression can activate the anti-ferroptosis rescue pathway.

Western blot of ACSL4 protein levels under TBHP-induced OA chondrocytes showed significantly up-regulated expression of this protein. However, IE-hAESCs-Exo intervention down-regulated the protein level of ACSL4, which was different from that of hAESCs-Exo intervention (Fig. 5D). To further validate the exact role of ACSL4 in chondrocyte ferroptosis, ACSL4 lentiviral vectors in chondrocytes were constructed for ACSL4 knockdown (sh-ACSL4) or control (sh-NC) (Fig. S2). ACSL4 knockdown led to significantly reduced ACSL4 expression in chondrocytes (Fig. 5E and F) and enhanced proliferation of chondrocytes (Fig. 5G and H). Moreover, sh-ACSL4 resulted in a decrease in COX2 levels but, conversely, an increase in the protein levels of FTH1, GPX4, SLC3A2, and SLC7A11, which are regulators known to inhibit ferroptosis (Fig. 5I). Additionally, sh-ACSL4 down-regulated the expression of MMP13 and ADAMTS5 while up-regulating ACAN and COL2A1 (Fig. 5J). Collectively, ACSL4 might attenuate OA progression by modulating ferroptosis in chondrocytes.

### Effects of ACSL4 overexpression on chondrocyte ferroptosis and aggravated OA

**Fig. 6.**
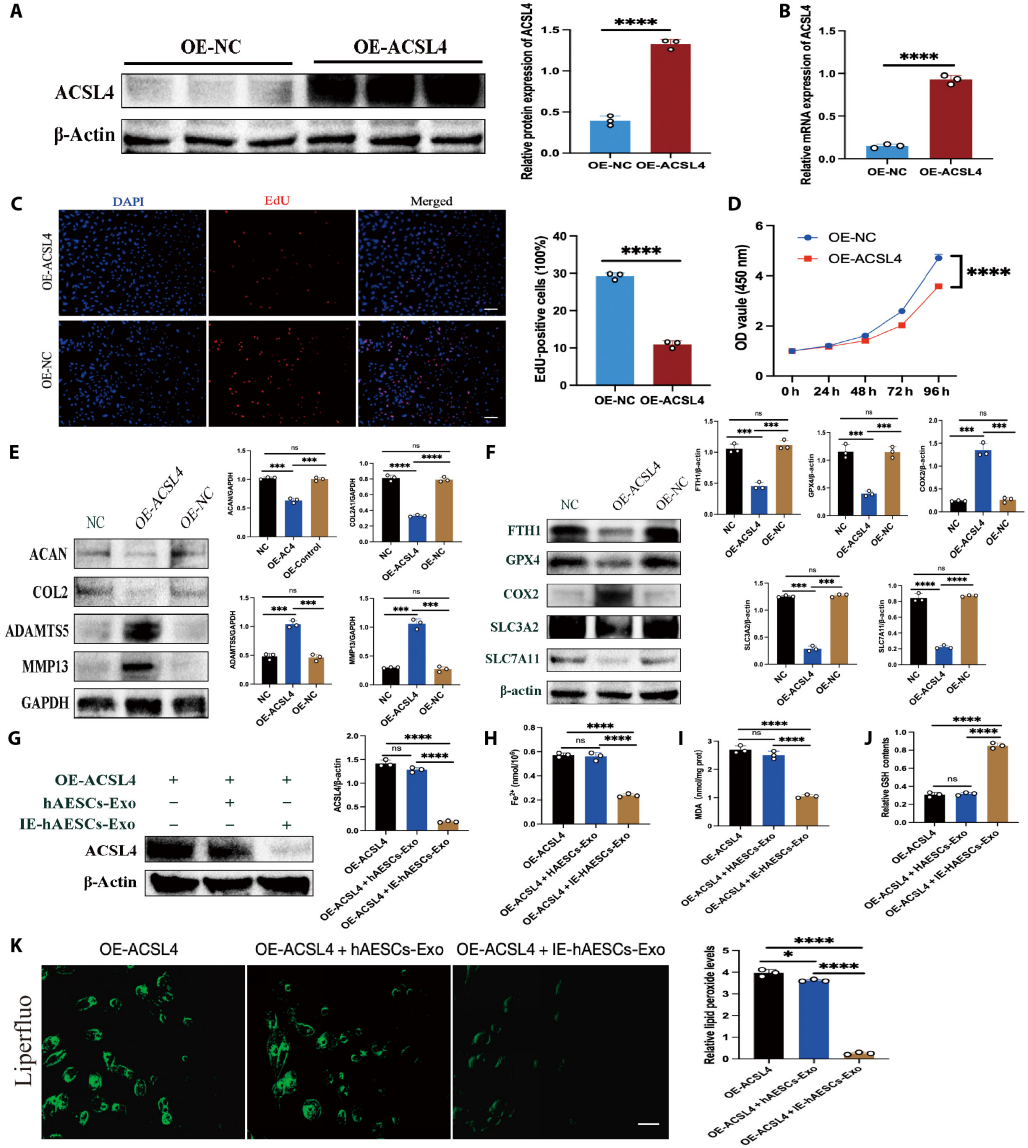
Effects of ACSL4 overexpression on chondrocyte ferroptosis and aggravated OA. (A) Western blot was employed to assess the efficiency and quantify the OE-ACSL4 transfection in chondrocytes (*n* = 3 per group). (B) qRT-PCR was utilized to evaluate the transfection efficiency of OE-ACSL4 (*n* = 3 per group). (C) EdU staining was conducted to assess chondrocyte proliferation and perform quantitative analysis following OE-ACSL4 virus transfection (*n* = 3 per group). Scale bar: 50 μm. (D) The CCK-8 assay measured the proliferation activity of chondrocytes post-OE-ACSL4 virus transfection. (E) Western blot analysis was used to detect changes in marker genes related to cartilage matrix metabolism and perform quantitative analysis (*n* = 3 per group). (F) Western blot analysis was conducted to examine the impact of OE-ACSL4 on ferroptosis-related marker genes and perform quantitative analysis (*n* = 3 per group). (G) Western blot analysis assessed the targeted regulatory effect and performed quantitative analysis of IE-hAESCs-Exo on ACSL4 (*n* = 3 per group). (H) Intracellular Fe^2+^ levels in chondrocytes posttreatment with IE-hAESCs-Exo and OE-ACSL4 were assessed using ferrous colorimetry (*n* = 3 per group). (I) MDA concentrations in chondrocytes were determined via MDA colorimetry (*n* = 3 per group). (J) GSH content was quantified using GSH colorimetry (*n* = 3 per group). (K) Lipid peroxidation fluorescence and its intensity in chondrocytes were analyzed using the Liperfluo probe (*n* = 3 per group). Scale bar: 20 μm. All data are presented as mean ± SD. Statistical significance was defined as *P* < 0.05 (*P < 0.05; **P < 0.01; ***P < 0.001; ****P < 0.0001).

An ACSL4-green fluorescent protein (GFP) lentiviral vector for ACSL4 overexpression (OE-ACSL4) and a GFP empty vector as a control (OE-NC) were constructed and enrolled for experiments to verify the relationship between ACSL4 and ferroptosis in chondrocytes (Fig. S3). Overexpression was confirmed via Western blot and quantitative real-time polymerase chain reaction (qRT-PCR) (Fig. 6A and B). OE-ACSL4 treatment resulted in inhibited chondrocyte proliferation (Fig. 6C and D and a significantly up-regulated protein level of COX2, a positive regulator of ferroptosis, and obviously down-regulated protein levels of FTH1, GPX4, SLC3A2, and SLC7A11, which inhibit ferroptosis (Fig. 6E). Additionally, OE-ACSL4 significantly up-regulated MMP13 and ADAMTS5 (markers of cartilage matrix catabolism) and down-regulated ACAN and COL2A1 (markers of chondrocyte ECM synthesis) (Fig. 6F). Further intervention in OE-ACSL4 chondrocytes with hAESCs-Exo and IE-hAESCs-Exo showed that IE-hAESCs-Exo significantly down-regulated ACSL4 protein expression, whereas hAESCs-Exo had no significant effect (Fig. 6G). In addition, IE-hAESCs-Exo markedly mitigated the OE-ACSL4-induced rise in intracellular Fe^2+^ and MDA levels (Fig. 6H and I). Conversely, IE-hAESCs-Exo treatment significantly reversed the reduction in GSH caused by OE-ACSL4 (Fig. 6J). Moreover, IE-hAESCs-Exo significantly inhibited the lipid peroxidation accumulation triggered by OE-ACSL4 (Fig. 6K). All of these results suggested that OE-ACSL4 might promote chondrocyte ferroptosis and exacerbate OA progression, whereas IE-hAESCs-Exo counteracted this adverse progression by inhibiting chondrocyte ferroptosis.

### lncRNA ACTA2-AS1 directly bound to ACSL4 and regulated its expression

**Fig. 7.**
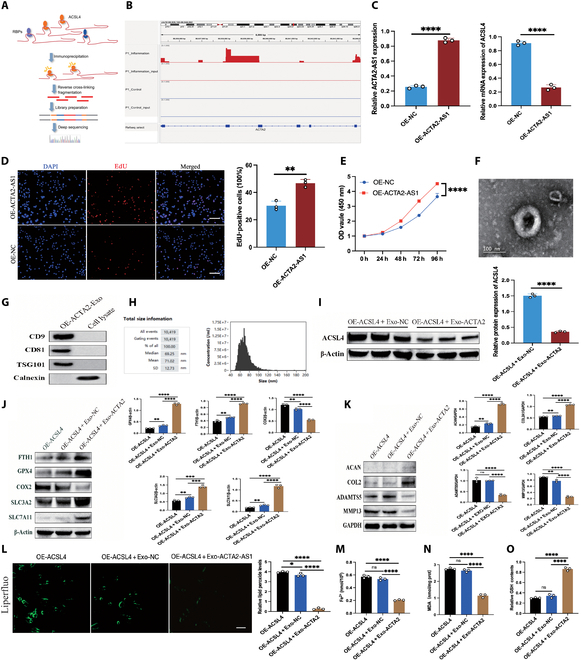
Long noncoding RNA (lncRNA) ACTA2-AS1 (actin alpha 2, smooth muscle antisense RNA1) directly bound to ACSL4 and regulated its expression. (A) Flowchart of RNA-immunoprecipitation and high-throughput sequencing (RIP-seq). (B) Identification of lncRNA with the highest binding affinity to ACSL4 using RIP-seq data. (C) qRT-PCR analysis of ACTA2-AS1 lentiviral transfection efficiency (*n* = 3 per group). (D) EdU staining and quantitative analysis to evaluate hAESC proliferation following ACTA2-AS1 viral transfection (*n* = 3 per group). Scale bar: 50 μm. (E) CCK-8 assay assessing hAESC proliferation post-ACTA2-AS1 viral transfection. (F) Transmission electron microscopy of ACTA2-Exo exosomes. Scale bar: 100 nm. (G) Western blot analysis of marker protein expression in ACTA2-Exo. (H) Nanoparticle size analysis of ACTA2-Exo. (I) Western blot and quantitative analysis of ACTA2-Exo effects on OE-ACSL4 (*n* = 3 per group). (J) Western blot analysis was conducted to assess ferroptosis following ACTA2-Exo intervention with OE-ACSL4, including quantitative analysis (*n* = 3 per group). (K) Western blot analysis evaluated the impact of ACTA2-Exo on cartilage matrix metabolism post-OE-ACSL4 intervention, with quantitative analysis (*n* = 3 per group). (L) The Liperfluo probe measured the fluorescence expression and intensity of lipid peroxidation in chondrocytes (*n* = 3 per group). Scale bar: 20 μm. (M to O) Changes in Fe^2+^, MDA, and GSH levels were observed after ACTA2-Exo intervention with OE-ACSL4 (*n* = 3 per group). All data are presented as mean ± SD. Statistical significance was defined as *P* < 0.05 (*P < 0.05; **P < 0.01; ***P < 0.001; ****P < 0.0001). RBPs, RNA-binding proteins.

Exo facilitate intercellular communication by transferring proteins, lipids, and lncRNAs. Therefore, lncRNAs interacting with ACSL4 were identified here using RNA immunoprecipitation followed by RIP-seq, which involved immunoprecipitating target proteins to capture associated RNAs (Fig. 7A). To elucidate the downstream molecular mechanisms of ACSL4 in protecting chondrocytes from ferroptosis, the dynamic interactions between ACSL4 and ferroptosis-related RNAs in vivo were examined using RIP-seq, with a purpose to clarify the role of ACSL4 in gene expression regulation. Our experiments revealed a strong association between ACSL4 and lncRNA ACTA2-AS1, inspiring our subsequent exploration of whether ACTA2-AS1 modulated ACSL4 expression (Fig. 7B). Furthermore, to verify the relationship between ACTA2-AS1 and ACSL4, we constructed an ACTA2-AS1-GFP lentiviral vector for overexpressing ACTA2-AS1 (OE-ACTA2-AS1) in hAESCs, using a GFP empty vector as a control (OE-NC) (Fig. S4). qRT-PCR confirmed successful transfection with the OE-ACTA2-AS1 lentivirus stably. Compared to the OE-NC group, the OE-ACTA2-AS1 group had significantly increased ACTA2-AS1 expression but decreased ACSL4 messenger RNA (mRNA) expression (Fig. 7C). Therefore, ACTA2-AS1 and ACSL4 mRNA might form a stable complex, regulating their functions through direct binding. CCK-8 and 5-ethynyl-2′-deoxyuridine (EdU) proliferation assays also indicated that the OE-ACTA2-AS1 group had enhanced hAESC proliferation (Fig. 7D and E). We then isolated and characterized Exo from the culture supernatant of hAESCs expressing OE-ACTA2-AS1, identifying ACTA2-Exo as an Exo-associated biomarker via TEM, comprehensive bio-nanoparticle analysis, and Western blot. All of these analyses confirmed the successful extraction and purification of Exo from OE-ACTA2-AS1-hAESCs (Fig. 7F to H). Subsequently, ACTA2-Exo was applied to chondrocytes expressing OE-ACSL4, resulting in a significant reduction in ACSL4 expression (Fig. 7I). Furthermore, ACTA2-Exo treatment in these chondrocytes led to a marked down-regulation of COX2 and increase in FTH1, GPX4, SLC3A2, and SLC7A11 protein levels (Fig. 7J). ACTA2-Exo also inhibited the expression of MMP13 and ADAMTS5 and obviously up-regulated ACAN and COL2A1 protein expression (Fig. 7K).

Reassessment through Liperfluo lipid peroxidation assay revealed that ACTA2-Exo significantly inhibited OE-ACSL4-induced lipid peroxidation accumulation (Fig. 7L). Moreover, ACTA2-Exo effectively mitigated the OE-ACSL4-induced increase in intracellular Fe^2+^ and MDA levels (Fig. 7M and N). Moreover, ACTA2-Exo treatment resulted in notable reverse of the reduction in GSH caused by OE-ACSL4 (Fig. 7O). In view of the above, ACTA2-AS1 could bind to ACSL4 and modulate its expression, and ACTA2-Exo could effectively alleviate OE-ACSL4-induced chondrocyte ferroptosis and OA progression.

## Discussion

This study unveils that the inflammatory milieu can modulate OA development by enhancing ACTA2-AS1 transcription in hAESCs. ACTA2-AS1 is transferred to chondrocytes via Exo secreted by hAESCs, where it inhibits ferroptosis by promoting the degradation of ACSL4, a key ferroptosis regulator. hAESCs outperform other types of stem cells in terms of convenient isolation, high abundance, ethical acceptability, non-immunogenicity, nontumorigenicity, etc. [33]. As evidenced by numerous existing publications [34–42], hAESCs exhibit potential in managing conditions such as neurodevelopmental disorders, spinal cord injuries, lung and chronic liver diseases, endocrine and metabolic disorders, renal injuries, cardiovascular diseases, wound healing, and uterine adhesions. As previously mentioned, hAESCs exhibit non-immunogenic properties due to the absence of human leukocyte antigen II on their surface. This characteristic prevents any immune response across different species, representing one of the most significant advantages of hAESCs. Furthermore, our animal experiments confirmed the absence of immune rejection. Stem-cell-mediated regeneration relies largely on the paracrine mechanism, with Exo carrying anti-inflammatory molecules, growth factors, and miRNAs. Exo have been recognized as a cell-free therapeutic solution, exhibiting low immunogenicity, which can ameliorate the pathogenic phenotype of chondrocytes [43–45].

So far, little is known about the effects of hAESCs-Exo on OA progression and treatment. This study examined the therapeutic potential of hAESCs-Exo through both in vitro and in vivo OA models. As previously described, the functionality of stem cells can be enhanced in specific inflammatory microenvironments. In our experiments, we utilized Exo derived from hAESCs that had been subjected to inflammatory pretreatment. Experimental results demonstrate that hAESCs under inflammatory conditions significantly enhance the functionality of their Exo. Notably, these Exo exhibit a superior ability to promote the repair of cartilage cells compared to those secreted by hAESCs in a standard environment. Consequently, intra-articular administration of hAESCs-Exo, IE-hAESCs-Exo in particular, significantly mitigated OA progression. Previous studies [46,47] have documented the role of inhibiting ferroptosis in reversing OA progression, offering a novel therapeutic approach. Accordingly, IE-hAESCs-Exo were employed in our experiments to target chondrocyte ferroptosis. It was observed to effectively inhibit both the onset and progression of chondrocyte ferroptosis and hence suppress OA. It should be acknowledged that there is an intrinsic interplay between ferroptosis and OA, presenting great challenges in this emerging field. Our prior proteomics analysis of inflammation-induced human OA chondrocytes, pre- and posttreatment with hAESCs-Exo, indicates significantly down-regulated ACSL4 following treatment. Additionally, pathway enrichment supported a notably inhibited ferroptosis pathway. Therefore, ACSL4 appeared to be pivotal in regulating lipid peroxidation and ferroptosis by activating polyunsaturated fatty acids (PUFAs) and orchestrating lipid metabolism reprogramming. The roles of ACSL4, a central driver of ferroptosis, hinge on its capacity to esterify PUFAs, which can produce a crucial substrate for lipid peroxidation by converting PUFAs to PUFA-CoA, and incorporate them into phospholipids [48–50].

Several existing studies have demonstrated the effect of ACSL4 on modulating ferroptosis and mitigating OA [51,52]. Consistently, the present study confirmed the function of ACSL4 in OA through overexpression and knockdown experiments in chondrocytes. Following ACSL4 overexpression, IE-hAESCs-Exo treatment further demonstrated its targeted therapeutic potential. However, further in-depth investigation is needed to get into the bottom of the interaction between IE-hAESCs-Exo and ACSL4. Exo contain various nucleic acids, such as mRNAs, noncoding RNAs, and miRNAs, which may have a role in disease progression through various mechanisms such as mediating inflammation, cell survival, and ECM deposition upon uptake by recipient cells [53–56]. In diverse cellular contexts and biological processes, lncRNAs can regulate gene expression at transcriptional and posttranscriptional levels, thus playing a significant role in cartilage ECM homeostasis and OA development [57–59]. Exo-derived lncRNAs are nanoscale vesicles capable of targeted drug delivery, which have been implicated in disease progression [60,61].

In this study, the interaction of lncRNAs with ACSL4 was identified, through RIP-seq, to investigate the interaction between IE-hAESCs-Exo and ACSL4. lncRNA ACTA2-AS1 was observed to bind to and function with ACSL4. At present, there is no report on the role of ACTA2-AS1 in ferroptosis and bone joint diseases, despite its known effects on promoting apoptosis and ameliorating hypoxic conditions in various diseases, including those of the liver, bile duct, colon, lung, cervix, and ovaries [62–66]. Our findings indicated that lncRNA ACTA2-AS1 bound to ACSL4, reducing its expression, which inhibited lipid peroxidation and regulated ferroptosis in chondrocytes. This regulatory effect is mediated through the delivery of IE-hAESCs-Exo, offering therapeutic potential.

Ferroptosis is primarily marked by lipid peroxide (LPO) accumulation, which is generally driven by lipid peroxidation and modulated by an oxidative-antioxidant system. The iron-dependent enzyme lipoxygenase initiates ferroptosis by generating lipid hydroperoxides, a process reliant on ACSL4-mediated lipid biosynthesis [67]. GPX4, a central regulator of ferroptosis, requires GSH, synthesized via the cysteine–glutamate antiporter SLC7A11, for exerting its function [68,69]. This study demonstrated the synergistic relationship between GPX4 and SLC7A11 following OE-ACSL4 and sh-ACSL4 in chondrocytes. IE-hAESCs-Exo potentially reverses ferroptosis by targeting multiple pathways.

Specifically, the delivery of Exo can restore GSH system function, neutralize lipid peroxidation products, enhance cystine uptake, maintain GSH synthesis, and down-regulate COX2, thereby inhibiting lipoxygenase activation in arachidonic acid metabolism. GPX4 is a key inhibitory of ferroptosis, which can regulate and restore its activity to eliminate LPOs, creating a positive feedback loop. This multi-level regulatory mechanism of IE-hAESCs-Exo in the ferroptosis of chondrocytes likely involves the modulation of iron metabolism, antioxidant defense, and lipid peroxidation signaling, effectively blocking cell death cascades. Additionally, Exo-mediated metabolic rebalancing extends to the regulatory network of ECM synthesis and decomposition.

## Conclusion

**Fig. 8.**
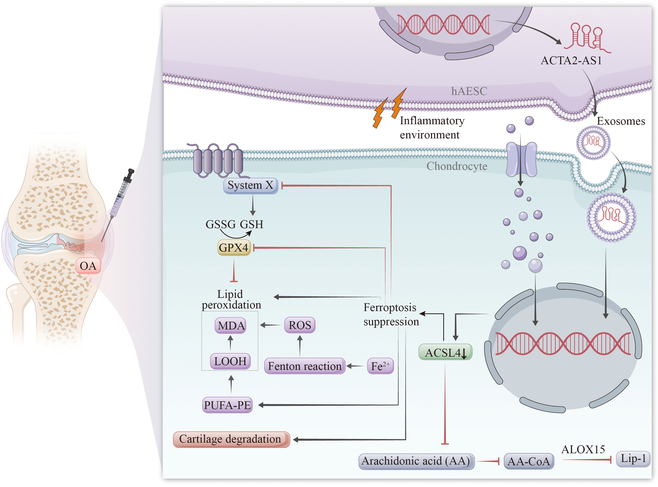
Graphical abstract. This study demonstrates that an inflammatory environment influences the onset and progression of OA by enhancing the transcription of ACTA2-AS1 in hAESCs. ACTA2-AS1 is transferred to chondrocytes via hAESC-derived exosomes, where it directly interacts with and down-regulates ACSL4 expression, thereby inhibiting lipid peroxidation. This mechanism modulates chondrocyte ferroptosis and mitigates cartilage degeneration. GSSG, glutathione disulfide; PUFA, polyunsaturated fatty acid.

In conclusion, ACSL4 is a critical factor in mediating the vulnerability of OA to ferroptosis. IE-hAESCs-Exo are identified as a novel mediator that inhibits lipid peroxidation by regulating ACSL4, thereby impeding chondrocyte ferroptosis; moreover, Exo can deliver ACTA2-AS1 to chondrocytes. Therefore, therapeutic strategies targeting ACSL4 may be promising in disrupting the pathological link between chondrocyte ferroptosis and OA. This study innovatively demonstrates an enhanced ACTA2-AS1 transcription in hAESCs under an inflammatory environment. hAESCs-Exo can further deliver ACTA2-AS1 to chondrocytes, where it directly binds to and down-regulates ACSL4, inhibiting lipid peroxidation. This regulation can mitigate ferroptosis in chondrocytes, alleviate cartilage degeneration, and ultimately influence OA progression (Fig. 8).

## Materials and Methods

### Ethics approval and consent to participate

Human cartilage samples were sourced from individuals undergoing total knee arthroplasty, and human amniotic tissue from discarded placentas post-cesarean section. To process human tissue samples, our study obtained official approval related to study protocols and procedures from the Ethics Committee of Northern Jiangsu People’s Hospital Affiliated to Yangzhou University (Approval No.: 2024ky252), ensuring compliance with the Declaration of Helsinki. Additionally, all animal experiments received approval from the Laboratory Animal Ethics Committee of Yangzhou University (Approval No.: 202407230).

### Isolation and characterization of hAESCs

P3 to P5 hAESCs were used for experiments in this study. After co-culture with respective induction media, the differentiation capacities of hAESCs into adipocytes, osteocytes, and chondrocytes were assessed through subsequent staining with alizarin red, alcian blue, and oil red O, respectively. The cell marker proteins of hAESCs were detected by flow cytometry (Beckman, CytoFLEX Flow Cytometer). In addition, the expression of SSEA-3, SSEA-4, and transcription factor OCT-4 was identified by immunofluorescence.

### Isolation and identification of Exo

After the collection of the supernatant from hAESCs, Exo were isolated via ultracentrifugation. Initially, differential centrifugation was applied to the supernatant (300g for 10 min, 2,000g for 20 min, and 10,000g for 40 min) to eliminate intact cells and debris. The supernatant was then concentrated 5 to 10 times using a 15-ml ultrafiltration centrifuge tube (Amicon Ultra-15) and filtered through a 0.22-μm membrane (Millipore, USA). Subsequent centrifugation was performed at 100,000g for 60 min at 4 °C to isolate protein-rich Exo. These Exo were resuspended in phosphate-buffered saline (PBS) and subjected to further centrifugation at 12,000g for 2 min to obtain crude Exo. After that, the supernatant was transferred to an Exosome Purification Filter (EPF column) for another centrifugation at 3,000g at 4 °C for 10 min. After that, purified Exo were yielded by collecting the liquid from the bottom of the EPF column. Finally, the ultrastructure of the Exo was identified using TEM (HT-7700, Hitachi, Japan). The particle size and marker antigens of the Exo were analyzed via nanoflow cytometry. In addition, the surface proteins CD9, CD81, TSG101, and calnexin were detected through Western blot.

### Exo labeling and chondrocyte uptake

The uptake of hAESCs-Exo by chondrocytes was assessed using PKH26-labeled hAESCs-Exo. These labeled Exo were co-incubated with chondrocytes at 37 °C for 24 h. Following incubation, unabsorbed Exo were removed by washing with sterile PBS. Eventually, the internalization of PKH26-labeled hAESCs-Exo (visible as red dots) within the chondrocytes was examined using inverted fluorescence microscopy (ZEISS).

### CCK-8 assay and Live/Dead assay

In accordance with the manufacturer’s instructions, the cytotoxicity and activity of the treated chondrocytes were assessed by CCK-8 (KTA1020, Abbkine) assay and calcein AM/propidium iodide staining (E-CK-A354, Elabscience), respectively. The optical density (OD) of the medium was measured at a wavelength of 450 nm using a microplate reader (Tecan, Switzerland), followed by the capture of suitable images by inverted fluorescence microscopy (ZEISS, Germany).

### Determination of intracellular Fe^2+^ levels

Chondrocyte iron ion detection was conducted using Cell Ferrous Iron Colorimetric Assay Kit (E-BC-K881-M, Elabscience). Briefly, a species with a strong absorption peak was produced at 593 nm after the binding of ferrous ions in the sample to the probe. The OD at this wavelength was linearly correlated with ferrous ion concentration within a specific range.

### Measurement of reduced GSH

GSH levels were quantified using a GSH assay kit (E-BC-K030-M, Elabscience) following the manufacturer’s protocol. Briefly, after the reaction of GSH with dithiodinitrobenzoic acid, thionitrobenzoic acid and glutathione disulfide were formed to generate a yellow compound. Then, the concentration of GSH was determined by measuring the OD at 420 nm using a microplate reader.

### MDA assay

With the use of an MDA colorimetric test kit (E-BC-K028-M, Elabscience), the MDA in the chondrocyte sample reacted with thiobarbituric acid (TBA) to form MDA–TBA compounds. After that, the MDA concentration was determined through the measurement of the OD value at 405 nm using an enzyme-linked immunosorbent assay reader.

### Assessment of lipid peroxidation by Liperfluo staining

Liperfluo, an analog of Spy-LHP, was employed to detect LPOs, as it is specifically oxidized by LPOs and exhibits strong fluorescence in organic solvents like ethanol. Chondrocytes were cultured in 24-well plates, followed by cell staining with Liperfluo (L248, Dojindo) for 30 min. Finally, the fluorescence intensity was assessed using an inverted fluorescence microscope (ZEISS, Germany).

### FerroOrange detection probe

FerroOrange is a novel fluorescent probe designed for imaging Fe^2+^ in living cells. Chondrocytes were cultured in 24-well plates, treated for 4 h, washed with PBS, and then stimulated in PBS at 37 °C with 5% CO_2_ for 20 min. Subsequently, the cells were stained with FerroOrange (F374, Dojindo) for 30 min and imaged using fluorescence microscopy (ZEISS, Germany).

### Intracellular ROS detection

H2DCFDA, a cell-permeable probe for detecting intracellular ROS, was utilized following a 4-h treatment of chondrocytes. The cells were stained with 10 μM H2DCFDA (HY-D0940, MCE) at 37 °C for 30 min. Subsequently, the samples were imaged using fluorescence microscopy (ZEISS, Germany) after washing 3 times with PBS.

### MMP detection

JC-1 is a widely used fluorescent probe for assessing MMP (∆Ψm). The MMP in chondrocytes was evaluated using fluorescence microscopy with JC-1 Mitochondrial Membrane Potential Assay Kit (E-CK-A301, Elabscience). Treated cells were incubated with JC-1 staining solution for 20 min at 37 °C and analyzed via fluorescence microscopy (ZEISS, Germany).

### Chondrocyte TEM

Cultured chondrocytes were discarded and fixed in 2.5% glutaraldehyde for 2 h at room temperature. The next step was 3-h fixation in 1% hydrochloric acid and 0.1 M phosphate buffer (pH 7.2) at room temperature. The samples were then gradually dehydrated in ethanol (30% to 100%) and acetone. Embedded samples were sectioned, with ultrathin sections collected on copper grids, followed by staining with uranium acetate and lead citrate. After that, the images were acquired using TEM at 80 kV (HITACHI HT 7800).

### Western blot

After the lysis of chondrocytes, protein concentration was measured via a bicinchoninic acid assay. Proteins were prepared by heating with sodium dodecyl sulfate–polyacrylamide gel electrophoresis loading buffer, separated on a 4% to 20% Bis-Tris gel, and transferred to a polyvinylidene fluoride membrane. Following membrane blocking, the next processing included overnight incubation with the primary antibody at 4 °C and then 1-h incubation with the secondary antibody at room temperature after washing. Subsequently, protein bands were visualized using a gel recording system and analyzed with ImageJ. The antibodies employed were anti-ACSL4 and anti-ADAMTS5 from Abcam and anti-ACAN, anti-COL2A1, anti-MMP13, anti-FTH1, anti-GPX4, anti-COX2, anti-SLC3A2, anti-SLC7A11, anti-GAPDH, anti-β-actin, and goat anti-rabbit IgG HL (horseradish peroxidase [HRP]) secondary antibody from ABclonal.

### Animal modeling and treatments

To investigate the effect of hAESCs on OA in vivo, 12-week-old male Sprague–Dawley rats were randomly assigned to receive various treatments. We established a DMM procedure to induce experimental OA models. Four weeks post-operation, the rats were divided into the sham group, vehicle group, and hAESC group for processing and observation. The hAESC group received injections of 1.0 × 10^6^ cells into the joint cavity 4 weeks after the establishment of the DMM model, with weekly injections maintained for a duration of 4 weeks. The sham and vehicle groups did not receive any treatment. Subsequently, the knee joint was harvested after the rat was euthanized for the subsequent experiment.

To investigate the therapeutic effects of hAESCs-Exo on OA in vivo, 12-week-old male Sprague–Dawley rats were randomly assigned to various treatment groups. An experimental OA model was established through knee DMM. Four weeks postsurgery, intra-articular injections were administered twice weekly for 6 weeks across different groups: sham, vehicle, normal saline (100 μl), hAESCs-Exo (5 × 10^10^ particles/ml, 100 μl), and IE-hAESCs-Exo (5 × 10^10^ particles/ml, 100 μl). Furthermore, a tracer experiment was conducted using hAESCs-Exo to assess Exo retention in the joint. Rats were anesthetized with intraperitoneal sodium pentobarbital. After anesthesia, rats were positioned in a live imaging system after shaving the knee area. The joint cavity was injected with 3 × 10^5^ PKH26-labeled hAESCs-Exo and IE-hAESCs-Exo (100 μl, single injection), respectively. In vivo fluorescence imaging of small animals using an in vivo 3-dimensional imaging system (IVIS Spectrum, PerkinElmer) was performed 2 h, 12 h, 1 d, 2 d, 3 d, 7 d, and 14 d after injection. Following imaging, rats were euthanized via cervical dislocation to excise and harvest their internal organs for further fluorescence analysis.

### Micro-CT analysis

The knee joints harvested from rats were subjected to fixation in 4% paraformaldehyde and imaging using micro-CT (NMC-200, NEMO) with a resolution of 15 μm, operating at 80 kV and 0.06 mA. Consistent thresholds were applied to evaluate each sample. At the same time of analyzing coronal and sagittal knee joint images, this experiment also calculated and compared parameters such as BV/TV, Tb.Pf, Tb.Sp, Tb.N, Tb.Th, and BMD.

### Histology staining and IHC

The harvested knee tissues underwent histopathological examination. Specimens were fixed in 4% paraformaldehyde and decalcified with EDTA for 30 d. Cartilage sections were stained using hematoxylin–eosin, and Safranin O/Fast Green. The effect of the treatment on osteoarthritic cartilage was evaluated using the OARSI scoring system. For IHC, cartilage sections were incubated with primary antibodies against ACAN, COL2A1, MMP13 (ABclonal), ACSL4 (Abcam), and GPX4 (ABclonal), followed by treatment using goat anti-rabbit IgG HL (HRP) secondary antibodies. Sections were then incubated with biotinylated secondary antibodies, counterstained with hematoxylin, and visualized using diaminobenzidine solution for IHC.

### Proteomics analysis

Before and after treatment with IE-hAESCs-Exo, proteomic sequencing was conducted on human OA chondrocytes to compare differential protein expression between the 2 conditions. Proteomics analysis was completed through 2 primary stages of mass spectrometry experimentation and data analysis. Firstly, the experimental phase involved protein extraction, peptide digestion, tandem mass tag labeling, chromatographic fractionation, and liquid chromatography–tandem mass spectrometry data acquisition, followed by database retrieval. Subsequently, database identification and quantitative analysis were performed using Mascot 2.2 and Proteome Discoverer 1.4. The next processing was protein cluster and enrichment analyses of the quantitative data from the target protein set.

### Lentiviral transduction

Lentiviral constructs for ACSL4-shRNA (sh-ACSL4), control shRNA (sh-NC), ACSL4 overexpression (OE-ACSL4), and control overexpression (OE-NC) were sourced from Genechem (Shanghai, China). The sh-ACSL4 sequence, GCAGCACAGACCTGCTTTAAG, was cloned into a GV493 vector. The OE-ACSL4 primers were Ubi-F, 5′-GGGTCAATATGTAATTTTCAGTG-3′, and FLAG-R-2, 5′-CCTTATAGTCCTTATCATCGTC-3′, cloned into a GV492 vector. Recombinant vectors were verified via DNA sequencing. Based on preliminary experiments, chondrocytes reaching a confluence of 30% to 50% were transfected using HitransG Transfection Reagent A (Genechem) according to the manufacturer’s instructions.

Similarly, lentiviral constructs for ACTA2-AS1 overexpression (OE-ACTA2-AS1) and control overexpression (OE-NC) were sourced from Genechem, Inc. (Shanghai, China). The OE-ACTA2-AS1 primer sequences were as follows: forward: 5′-AGGTCGACTCTAGAGGATCC-3′; reverse: 5′-GTGCTTAGGCACTGCAGTTG-3′. These sequences were cloned into a GV513 vector, and the recombinant vector was confirmed via DNA sequencing. As supported by the preliminary experiments, hAESCs at 30% to 50% confluence were transfected using HitransG Transfection Reagent P (Genechem) as instructed. Finally, the transfection efficiency was assessed by qRT-PCR for subsequent experiments.

### Quantitative real-time PCR

After total RNA extraction from cells using RNA Easy Fast Cellular RNA Extraction Kit (DP451, TIANGEN), complementary DNA (cDNA) was synthesized with cDNA Synthesis Kit (KR118, TIANGEN). Real-time PCR was conducted using Fast Real-Time PCR System (FP217, TIANGEN), with β-actin serving as an internal control. Gene expression was quantified via the 2^−ΔΔCt^ method. Details of relevant primer sequences are summarized in Tables S1 and S2.

### EdU staining

EdU staining was performed using an EdU kit (E-CK-A377, Elabscience) as instructed to evaluate cell proliferation post-virus transfection. Upon reaching suitable confluence, cells were switched to EdU medium and incubated at 37 °C with 5% CO_2_. Cells were then fixed with 4% paraformaldehyde and treated with Click reaction solution for 30 min at room temperature. After washing with PBS containing 3% bovine serum albumin, cells were stained with 4′,6-diamidino-2-phenylindole (DAPI) and examined via fluorescence microscopy. The proliferation rate was determined by calculating the ratio of EdU-positive cells to total DAPI-positive cells.

### RNA-immunoprecipitation and high-throughput sequencing

The capture of protein-bound RNA in vivo was realized through immunoprecipitation of the target protein, followed by the high-throughput sequencing of the RNA. This method enabled the elucidation of the molecular mechanism of gene expression regulation by ACSL4 via mapping its binding patterns with numerous RNA targets and quantifying binding intensity. In addition, call peak analysis of BAM files provided enriched region data, peak distribution across gene functional elements, and motif prediction, identifying peak-enriched lncRNA.

### Statistical analysis

Experimental results were replicated a minimum of 3 times, with data expressed as mean ± standard deviation. Statistical analyses were conducted using GraphPad Prism 10.2.0. A 2-tailed Student *t* test was employed for 2-group comparisons. One-way or 2-way analysis of variance followed by Tukey’s post hoc test was used for multigroup comparisons. Statistical significance was defined as *P* < 0.05 (*P < 0.05; **P < 0.01; ***P < 0.001; ****P < 0.0001).

## Data Availability

The data are freely available upon request.
