# Supplementary material for: Human Amniotic Epithelial Stem Cell Exosomes Regulate Chondrocyte Ferroptosis through ACTA2-AS1-Targeted Binding to ACSL4 for Osteoarthritis Intervention
Source: Research (Wash D C). 2025 Aug 8;8:0814. doi: 10.34133/research.0814 (PMC12332261; doi:10.34133/research.0814)
Supplement: Supplementary 1 — Figs. S1 to S4 Tables S1 and S2 [file research.0814.f1.docx]

Supplementary Materials for

**Human amniotic epithelial stem cell exosomes regulate chondrocyte ferroptosis through ACTA2-AS1 targeted binding to ACSL4 for osteoarthritis intervention**

Xiaofei Wang^1,2,3,4*^, Zhimin Wu^1,2,3*^, Lei Xu^2,3^, Linbing Lou^2,3^, Yuxia Yang^2,3^, Jian Zhang^2,3^, Haixiang Miao^2,3^, Cunyi Xia^2,3^, Zhiwei Peng^1,2,3^, Dongsheng Yang^5^, Zhiwen Tao^2,3^, Xiangji Meng^1,2,3^, Wenkang Liu^2,3^, Meijuan Yuan^2,3^, Jingcheng Wang^2,3,6#^, Wenyong Fei^2,3,6#^, Jihang Dai^2,3,6#^

**This PDF file includes:**

**Supplementary Figures 1-4**

**Supplementary Tables 1-2**

**
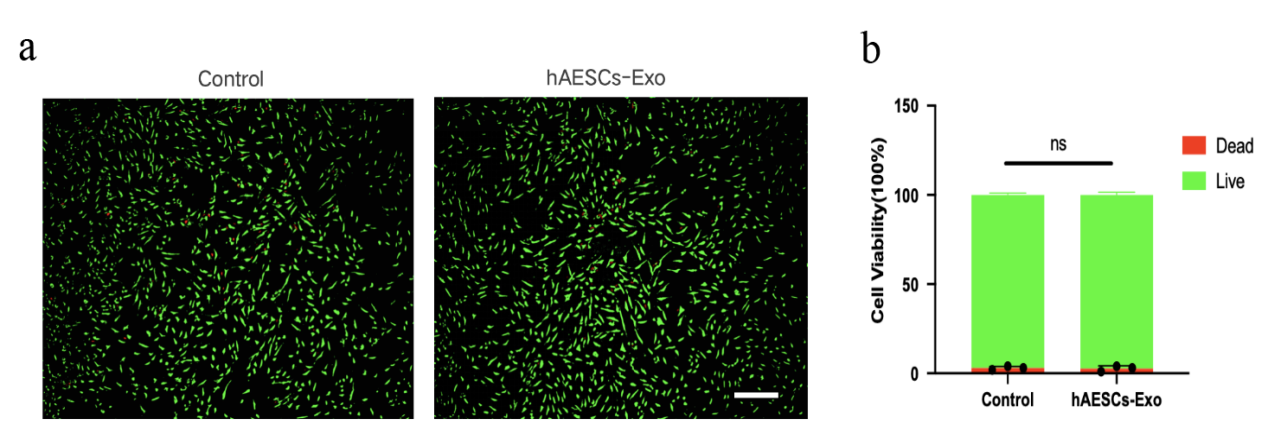
**

**Supplementary Figure 1. Live/dead cell assay with double staining to assess exosome toxicity.** The biosafety of exosomes on chondrocytes was evaluated using live/dead double staining. **a** Live cells were indicated in green, while dead cells were indicated in red (n=3 per group). Scale bar: 100 μm. **b** The statistical analysis of cell numbers showed that the viability of chondrocytes was sustained without a significant decrease after 24 hours of hAESCs-Exo treatment. These findings demonstrate that hAESCs-Exo did not exhibit significant cytotoxic effects on chondrocytes, confirming its biosafety. All data are presented as the mean ± SD. ns：P > 0.05.


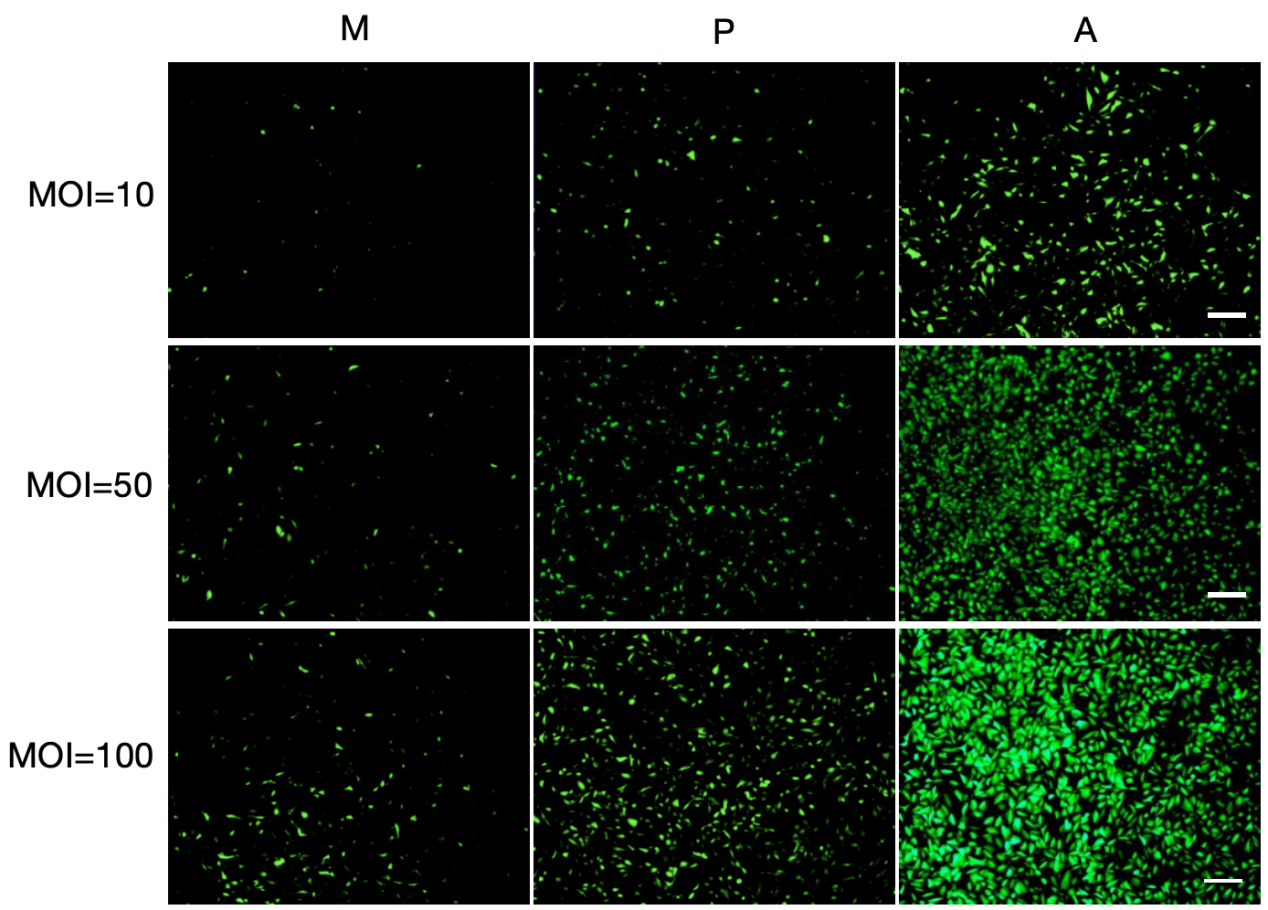


**Supplementary Figure 2. Results of the sh-ACSL4 lentivirus transfection pre-experiment.** To elucidate the role of ACSL4 in OA progression, we developed a chondrocyte model with ACSL4-specific knockdown (sh-ACSL4). Preliminary experiments determined an optimal infection efficiency of approximately 80%. We identified that an MOI of 50, with a viral concentration of 5×10^7^ TU/mL, combined with Normal+HiTransG A liquid, provided the best transfection enhancement. This setup minimized viral load while maintaining cell viability and transfection efficiency, aligning with our experimental design criteria. Scale bar: 100 μm.

**
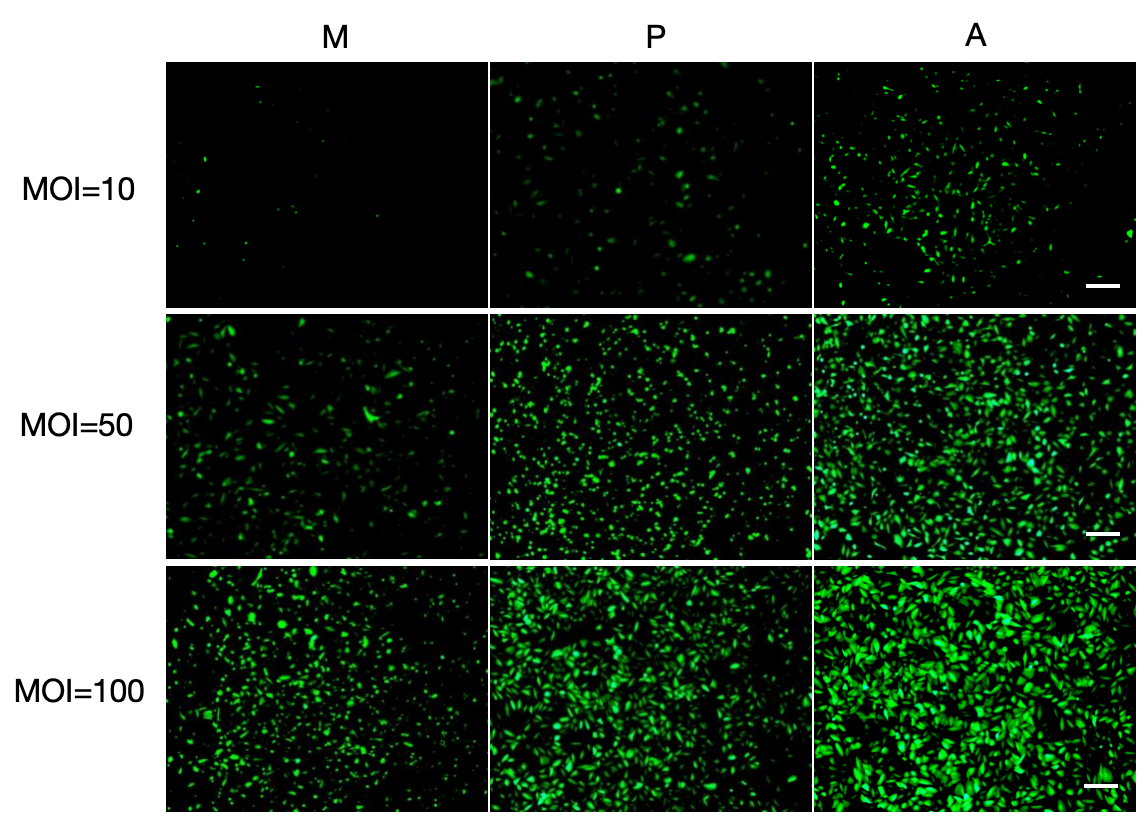
**

**Supplementary Figure 3. Pre-experimental results of OE-ACSL4 lentivirus transfection of chondrocytes.** To further explore the role of ACSL4, we developed a chondrocyte model with ACSL4 overexpression (OE-ACSL4) for validation. In preliminary experiments, we determined an optimal infection efficiency of approximately 80%. The most effective transfection, requiring minimal viral load, was achieved with a MOI of 50 and a viral concentration of 5×10^7^ TU/mL. The addition of Normal+HiTransG A solution enhanced transfection efficiency, and both cell growth and transfection efficiency aligned with the experimental design criteria. Scale bar: 100 μm.

**
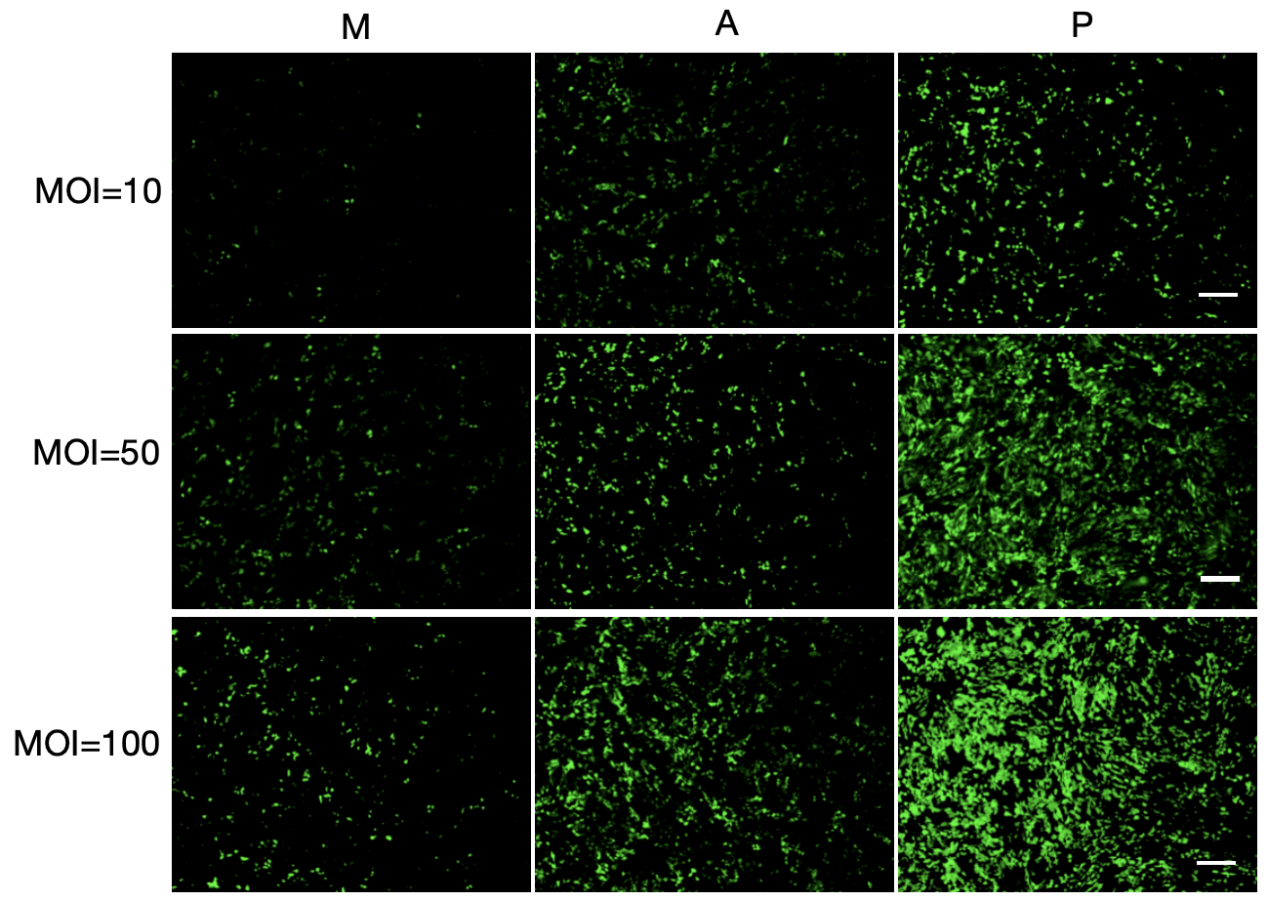
**

**Supplementary Figure 4. Pre-experimental results of OE-ACTA2-AS1 lentivirus transfection of hAESCs.** To elucidate the biological role of ACTA2-AS1, we developed an ACTA2-AS1 overexpression model (OE-ACTA2-AS1) in hAESCs. In preliminary experiments, we determined an optimal infection efficiency of approximately 80%. We achieved this with an MOI of 50 and a viral concentration of 5×10^7^ TU/mL, supplemented with Normal+HiTransG P solution to enhance transfection. This approach ensured optimal viral usage while maintaining satisfactory cell growth and transfection efficiency, aligning with our experimental objectives. Scale bar: 100 μm.

**Supplementary Table 1. PCR primers for gene editing efficiency assay.**

| **Primer** | **Sequence (5'-3')** |
| --- | --- |
| ACSL4 | （F）CTTGAAGTGAATCGCAGAGTGAATAAC |
|  | （R）GGTCTCACAGAAGATGGCAATGG |
| β-actin | （F）GGTTCCGCTGCCCTGAGG |
|  | （R）GGAGTTGAAGGTAGTTTCGTGGATG |

**Supplementary Table 2. PCR primers for gene editing efficiency assay.**

| **Primer** | **Sequence (5'-3')** |
| --- | --- |
| ACTA2-AS1 | （F）GTTCTGGAGGCTTGATATGG |
|  | （R）TCCTTCATCGGTAGGCAACAAAC |
| β-actin | （F）GGTTCCGCTGCCCTGAGG |
|  | （R）GGAGTTGAAGGTAGTTTCGTGGATG |
